# Relationships among Safety Climate, Safety Behavior, and Safety Outcomes for Ethnic Minority Construction Workers

**DOI:** 10.3390/ijerph15030484

**Published:** 2018-03-09

**Authors:** Sainan Lyu, Carol K. H. Hon, Albert P. C. Chan, Francis K. W. Wong, Arshad Ali Javed

**Affiliations:** 1 Department of Building and Real Estate, The Hong Kong Polytechnic University, 181 Chatham Rd. South, Hung Hom, Kowloon, Hong Kong, China; albert.chan@polyu.edu.hk (A.P.C.C.); francis.wong@polyu.edu.hk (F.K.W.W.); 2 School of Civil Engineering and Built Environment, Queensland University of Technology (QUT), Gardens Point, Brisbane, QLD 4001, Australia; carol.hon@qut.edu.au; 3 School of Economics and Finance, Massey University, Private Bag 11 222, Palmerston North 4442, New Zealand; a.a.javed@massey.ac.nz

**Keywords:** construction safety and health, ethnic minority construction workers, safety climate, safety behavior, safety outcome

## Abstract

In many countries, it is common practice to attract and employ ethnic minority (EM) or migrant workers in the construction industry. This primarily occurs in order to alleviate the labor shortage caused by an aging workforce with a lack of new entrants. Statistics show that EM construction workers are more likely to have occupational fatal and nonfatal injuries than their local counterparts; however, the mechanism underlying accidents and injuries in this vulnerable population has been rarely examined. This study aims to investigate relationships among safety climate, safety behavior, and safety outcomes for EM construction workers. To this end, a theoretical research model was developed based on a comprehensive review of the current literature. In total, 289 valid questionnaires were collected face-to-face from 223 Nepalese construction workers and 56 Pakistani construction workers working on 15 construction sites in Hong Kong. Structural equation modelling was employed to validate the constructs and test the hypothesized model. Results show that there were significant positive relationships between safety climate and safety behaviors, and significant negative relationships between safety behaviors and safety outcomes for EM construction workers. This research contributes to the literature regarding EM workers by providing empirical evidence of the mechanisms by which safety climate affects safety behaviors and outcomes. It also provides insights in order to help the key stakeholders formulate safety strategies for EM workers in many areas where numerous EM workers are employed, such as in the U.S., the UK, Australia, Singapore, Malaysia, and the Middle East.

## 1. Introduction

For many years it has been common practice to attract and employ ethnic minority (EM) workers in the construction sector. EM construction workers are of great importance to many countries, as evidenced by their relatively high and increasing proportions in the total construction workforce. For example, EM construction workers account for approximately 69% of the total construction workforce in Malaysia [1], 20% in Australia [2], and 30% in Spain [3]. In Hong Kong, EMs refer to persons who are of non-Chinese ethnicity, as defined in the 2011 Population Census Thematic Report: Ethnic Minorities [4]. The number of EM construction workers has increased significantly in Hong Kong and is anticipated to grow continually due to aging and shortages in the construction workforce [5].

With the increasing proportion of EMs in the construction industry, their safety and health issues have drawn growing worldwide attention [6,7]. Numerous studies have shown that EM construction workers are more vulnerable to injuries and fatalities. Tutt et al. [7] pointed out that EM construction workers in the UK face a relatively higher risk and are more vulnerable to fatal and non-fatal injuries compared with local workers. According to the statistics, EM workers in the UK accounted for 17% of construction deaths during 2007–2008, while only representing 2.4–8% of the construction workforce [8]. In the U.S., Hispanic workers also suffer a disproportionately higher accident rate than non-Hispanic workers [9]. Hispanic workers made up 25.5% and 27.3% of total construction workforce in 2012 and 2013, respectively, but accounted for 27.4% and 29.1%, respectively, of the total fatalities [10,11]. The situation is similar in Hong Kong. Due to lack of official statistics, an investigation of local newspaper archives from 2000 to 2005 was carried out by the research team to determine the number of fatalities of EM construction workers. The results show that the proportion of fatalities of EM construction workers with respect to the total construction fatalities is at least 6.2% (considering that some fatal accidents may not be reported) whereas they accounted for 1.5% of the total construction workforce [12].

Safety climate has been found to be a significant predictor of safety performance by many studies [13,14,15,16,17,18]. Unsafe activity on the part of the workers is a main contributing factor to accidents [18,19]. Compared with traditional safety measurements, safety climate is regarded as more proactive and sensitive in terms of providing safety-related information [20] and predicting safety behavior and safety outcomes [21]. The benefits of development and evaluation of safety climate are manifold. These benefits include: (1) the diagnosis of safety management deficiencies [22]; (2) provision of internal and external benchmarking [20,23]; (3) focus on safety efforts to improve specific areas [23]; and (4) optimization of safety-related time and resources [24]. Although a great number of studies have stressed the effects of the safety climate on local workers in the construction industry, research has not focused specifically on the effects for EM construction workers. The safety practices of EMs may differ from those of their local counterparts, due to cultural and religious disparities [25,26,27,28,29], language barriers [27,28,30], lack of appropriate safety training and safety knowledge [31], and risky and hazardous assigned tasks [24]. For instance, regarding the influence of national culture on safety, Mearns and Yule [32] revealed that masculinity and power distance have a significant impact on risk-taking behaviors, and Mohamed et al. [33] found that national culture can influence the safety awareness and beliefs of workers. The workers with religious beliefs were found to have a more positive perception of safety climate, as the concept of care and emphasis on values in most of major religions contribute to cultivating a good safety climate [34].

This study aims to determines the relationships among safety climate, safety behaviors, and safety outcomes for EM construction workers, and provide recommendations for improving the safety and health of this population. This study is part of a broad range of research on the occupational safety and health of EMs in Hong Kong. This research contributes to filling the research gap on safety climate regarding EMs by providing the empirical evidence of mechanisms underlying the relationships between safety climate and safety performance for EM construction workers. The findings of this research will help both researchers and professionals to have a deeper understanding of the determinants of safety, and offer effective incentives to improve the safety performance of EMs.

## 2. Literature Review and Research Hypotheses

### 2.1. Safety Climate

Drawing on studies of organizational climate, Zohar firstly defined the term safety climate [35]. Since then, substantial attention has been paid to the study of safety climate, especially by psychosocial and safety researchers, e.g., developing a safety climate instrument, determining safety climate structure, and analyzing effects of safety climate on other safety-related variables. Many studies have identified safety climate factors across various industries [17,36,37]. Nevertheless, there is a lack of consensus on the number and structure of safety climate factors. Some review studies attempted to obtain common or core safety climate factors through either qualitative or quantitative approaches. For instance, Seo et al. [24] captured five common factors, including: management commitment, supervisor support, coworker support, employee participation, and competence level. Olsen [38] identified five common cross-industrial safety climate constructs, consisting of: organizational management support of safety; transitions and teamwork across units; supervisor/manager expectations and actions promoting safety; learning, feedback, and improvement; and teamwork. Wu et al. [39] tested and validated the common safety climate factors identified through a literature review, and finally obtained five core factors (i.e., safety priority; safety supervision, training, and communication; safety rules and procedures; and safety involvement). However, disparities and divergences still exist in common safety climate dimensions identified from these review studies. They may be due to the difference in sample and context, arbitrariness of naming factors, and differences in measurement instruments. Although consensus on the dimensions of safety climate has not been reached, the usefulness of safety climate in predicting safety-related outcomes is widely recognized in both developing and developed countries.

### 2.2. Safety Behavior and Safety Outcome

Safety behaviors are actual behaviors that individuals perform at work [40]. Based on Borman and Motowidlo’s work performance typology [41], Neal et al. [42] differentiated safety behaviors into safety participation and safety compliance. These two dimensions have been adopted by many following safety behavior-related studies, such as Lu and Yang [43] and Vinodkumar and Bhasi [44]. Safety participation refers to frequent voluntary behaviors that are not likely to promote the personal safety directly but contribute to improving safety in workplace, such as attending meetings and helping coworkers [45]. In contrast, safety compliance denotes mandated behaviors that should be conducted to maintain the safety of workplace, such as wearing personal protective equipment and complying with safety rules and procedures [42,45].

Compared with safety behavior, safety outcomes are tangible and organizational measurements, including for example near-misses, injuries, and fatalities [40]. Previous safety research studies have used statistical accident and injuries data for measuring safety outcomes of workers. Recent studies have used alternative tools such as self-reported injury data [15,17,46]. Accident and injury data collected through the questionnaires could be considered as reliable and valid [47].

### 2.3. Relationships among Safety Climate, Safety Behavior, and Safety Outcome

The significant influence of safety climate on accident and injuries has been demonstrated by many empirical studies [14,15,16,24]. These findings indicate that the higher the level of safety climate, the lower the accident rate. This relationship between safety climate and injuries was mediated by employee’s behaviors [48]. Safety climate was anticipated to positively affect the safety behaviors of employees. As a result of reward or social exchange theory, a positive safety climate can foster and promote safety behaviors [42,49,50,51]. However, some studies did not find any relation between the safety climate and safety behaviors [37,52]. This study has taken the positive findings into consideration and expected that safety climate has a positive influence on safety behaviors of EMs. In addition, Neal and Griffin [45] found that safety climate played a lagged role in safety motivation, and safety motivation had a lagged effect on safety participation instead of safety compliance. Thus, it would be expected that safety participation would be more greatly influenced by safety climate than safety compliance of EMs. The following hypotheses in the terms of the relationship between safety climate and safety behaviors of EMs are proposed:

**Hypothesis** **1.** Safety climate will have a positive effect on the safety compliance of EM construction workers.

**Hypothesis** **2.** Safety climate will have a positive effect on the safety participation of EM construction workers.

**Hypothesis** **3.** The relationship between safety climate and safety participation will be stronger than that between safety climate and safety compliance.

Meanwhile, decent safety behaviors could lead to good safety outcomes. Improved safety behaviors can contribute to reducing the number of near-misses and injuries [51]. A higher level of safety compliance will likely reduce the number of accidents and injuries resulting from violating safety rules and procedures. A higher level of safety participation will likely reduce the number of accidents and injuries through better engagement of employees in safety activities and helping co-workers. Compared with safety compliance, safety participation is anticipated to have a stronger relationship with safety outcomes. The hypotheses related to the connections between safety behaviors and safety outcomes were proposed as follows:

**Hypothesis** **4.** Safety participation will negatively influence near-misses and injuries of EM construction workers.

**Hypothesis** **5.** Safety compliance will negatively influence near-misses and injuries of EM construction workers.

**Hypothesis** **6.** The relationship between safety compliance and near-misses and injuries will be stronger than that between safety participation and near-misses and injuries.

### 2.4. The Research Model

**Figure 1:**
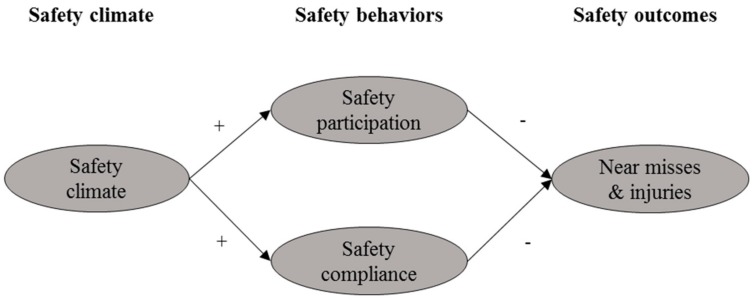
Initial theoretical model and research hypotheses.

Based on the literature, a theoretical research model was developed to explore the relationships among EMs’ perceptions of safety climate, safety participation, safety compliance, and near-misses and injuries (shown in Figure 1). In this study, safety climate, safety participation, safety compliance, and safety outcomes are latent variables, which cannot be directly observed and measured. The safety climate factors of EM workers in this study were determined in Chan et al. [53]. They are F1: *Safety management commitment, safety resources, and safety communication*; F2: *Employee involvement and workmate’s influence*; and F3: *Safety rules, procedures and risks*. For further details, please refer to Chan et al. [53].

## 3. Research Methods

After the research model was proposed based on the literature review, a questionnaire instrument was designed to collect data from respondents.

### 3.1. Questionnaire Instrument Design

In order to minimize instrumentation threat, a three-part questionnaire was developed based on the previous studies, where the validity and reliability of the construct were verified (see Table 1). In part A, respondents’ personal information was collected, including working level, age, gender, marital status, education level, working experience in the construction industry, and other related personal information.

In part B, EM workers’ perceptions about safety climate were gauged. This study employed the Safety Climate Index (SCI) of the Occupational Safety and Health Council in Hong Kong to measure safety climate. As reported in Chan et al. [53], three safety climate factors encapsulating 16 measurement items were determined to be fit for safety climate measurement for EM workers. Using a second-order safety climate model, three safety climate factors consisting of 16 questions were identified (see Appendix A). A five-point Likert scale was used for data collection about safety climate (where 1 = strongly disagree, 2 = disagree, 3 = neutral, 4 = agree and 5 = strongly agree).

Part C solicits data of safety participation, safety compliance, near-misses and occupational injuries of EMs. Two statements were adopted from Neal and Griffin [45] (p. 953) and Hon et al. [12] (p. 18) to measure the safety participation of the respondents with a 5-point ordinal scale (where 1 = never; 2 = yearly; 3 = monthly; 4 = weekly; and 5 = daily). Two questions were used from Mohamed [54] (p. 383) and Hon et al. [17] (p. 18) to measure the safety compliance of the respondents and their co-workers in terms of time (0–100). Four questions were asked to capture near-misses and occupational injuries of EM workers during the past 12 months with a 5-point ordinal scale (where 1 = never; 2 = 1 time; 3 = 2–3 times; 4 = 4–5 times; and 5 = over 5 times). Usually 12-month reference is frequently used in accident surveys to obtain an adequate number of accidents for analysis [17,55]. Measures of safety behaviors and safety outcomes can be found in Appendix A.

### 3.2. Respondents and Procedures

The questionnaire was administered to EMs working in civil and building projects in Hong Kong. The questionnaire was initially designed in English and Chinese. For the convenience of EM frontline workers, the questionnaire was then translated into Nepali (for Nepalese workers) and Urdu (for Pakistani workers), as these two ethnic groups are representing the highest proportion of non-Chinese male construction workers in Hong Kong (23.2% and 18.9%, respectively) [4]. Formal ethics approval was obtained from the researchers’ institution. A pilot study was conducted with 6 academic staff, 4 industrial practitioners, and 10 EM construction workers, following which modifications were made to the format and description of several items.

Initially 82 companies were invited through the researchers’ industrial links, and finally eight contactors and 12 sub-contractors who employed numerous EMs agreed to participate in this survey. This nonrandom sampling method is suitable to this study as there is no available list of EM construction workers and their employers. The low participation rate of contractors and subcontractors in this research is probably because most of the companies invited did not employ EM workers or did not have a sufficient number of EM workers to qualify for participation in this research. The participation rate of all companies is around 24%. The low participation rate of contractors and subcontractors in this research is because most of the companies invited did not employ EM workers or did not have a sufficient number of EM workers to be qualified to participate in this research. However, around 74% of companies that employed EMs participated in this survey. Thus, the 20 companies who participated in this research form a reasonably representative sample of the companies employing EM workers in Hong Kong.

Prior to each construction site visit, the researchers communicated with management (e.g., site manager, safety officers, and supervisor) regarding selection criteria of EM workers (i.e., non-Chinese ethnicity and engaged in construction works) and process of questionnaire survey through face-to-face discussions and telephone conversation. In total, 15 construction sites were assigned by management and the number of EMs working on these construction sites ranged from around 40 to 70. To avoid clustering within specific construction sites, the employers were advised to randomly select approximately 30 EM workers from the roster at each site to participate in this survey. In addition, some measures were taken to ensure a high response rate of EM respondents for producing a valid and reliable result. Firstly, the researchers visited the majority of the targeted construction sites to collect data face-to-face. Nulty [56] found that face-to-face administration can contribute to a higher response rate. Secondly, during each site visit, the surveyors who could speak the native language of the EMs explained the research objective and clarified queries to EMs in their native languages (e.g., explanation of some questions). All respondents were informed that the survey was anonymous and the collected data would be analyzed and reported collectively for academic purpose. Management was not present during the data collection process with EM workers onsite to avoid undue influence and bias. The EM participants took an average of 40 minutes to complete the questionnaire.

In total, 450 questionnaires were distributed and 349 of them were collected from managers, supervisors, and frontline workers. The response rate of this questionnaire survey was 77%, which was considered satisfactory by Moser [57]. Twenty-nine questionnaires were discarded and 320 valid questionnaires remained. These 29 questionnaires were found to be invalid due to a significant amount of missing or incomplete data (missing data >10%) and a very high proportion of same answers. The missing data from questionnaires completed by EMs mainly resulted from time constraints. There was a small proportion of EM workers who had low reading proficiency in even their own language, leading to their difficulties in filling in all questions. They only answered part of the questions. The missing data points are a random subset of the data. Since the aim of the research is to explore the safety of EM workers, only 289 completed questionnaires of frontline workers were selected for further analysis.

### 3.3. Data Analysis

Quantitative data collected from 289 questionnaires were firstly analyzed using Statistical Package for the Social Sciences (SPSS ver. 20.0, Chicago, IL, USA). Given that the safety climate questionnaire was a mix of positive and negative statements, the values of negative statements were reversed to corresponding positive values before checking the reliability. For example, score “5” of the question B1 “Productivity is usually seen as more important than health and safety by the company” (Likert scale of 1 to 5) was changed to “1” because a lower ranking of this attribute shows a high level of the safety climate. Structural equation modelling (SEM) was employed to validate the constructs and test the hypothesized model in Analysis of Moment Structures (AMOS ver. 20.0, IBM, Armonk, NY, USA) against the empirical data set. The SEM technique has been adopted by many researchers in the field of construction management. It combines the factor analysis and path analysis to test the measurement of constructs and causal relationship between latent variables [58].

## 4. Results

### 4.1. Descriptive Statistics Analysis

Table 2 lists the demographic information of respondents, including nationality, gender, age, employer, education level, work experience in the construction sector, and length of service with the current company. Nearly 80.6% (N = 233) of respondents were Nepalese workers, and 19.4% (N = 56) were Pakistani workers. The mean values of F1, F2, and F3 of safety climate were 3.81, 3.98, and 3.28, as shown in Table 3. As for safety behaviors of EMs, the mean value of safety compliance (Mean = 4.32) was higher than that of safety participation (Mean = 3.64). 

### 4.2. Reliability Analyses

Since the questionnaire instrument incorporates several constructs, the internal consistency of each construct needs to be evaluated. Cronbach’s alpha reliability test was carried out on the survey data to measure internal consistency [59]. A Cronbach’s alpha coefficient of a scale above 0.70 is considered to “suffice” for the research [59]. The values of Cronbach’s alpha are tabulated in Table 3. Cronbach’s alpha values of six constructs were all above 0.7, except for the construct of F3. Cronbach alpha values are quite sensitive to the number of items in the scale. If the Likert scale has fewer than ten items, low Cronbach values (e.g., 0.50) are expected [60].

The inter-item and item-total correlation matrices were also examined to assess the internal consistency. The inter-item correlation evaluates the relationships among items [61], while the item–total correlation measures the relationship between one item and the total items [62]. The percentage of item–item correlation coefficients ranging within 0.20–0.70 was greater than 50% (threshold for acceptance) [63] and item–total correlation values were all greater than 0.30 (threshold for acceptance) [59].

### 4.3. Testing of the Structural Model

SEM was conducted to test the proposed hypotheses and the aforementioned research model. The significance level was set as 0.001. Goodness-of-fit indices help to assess if the hypothesized model fits the empirical data. Three categories of goodness-of-fit measures were adopted, including preliminary fit criteria, overall model fit, and fit of internal structural model, as shown in Table 3 [64].

**Figure 2:**
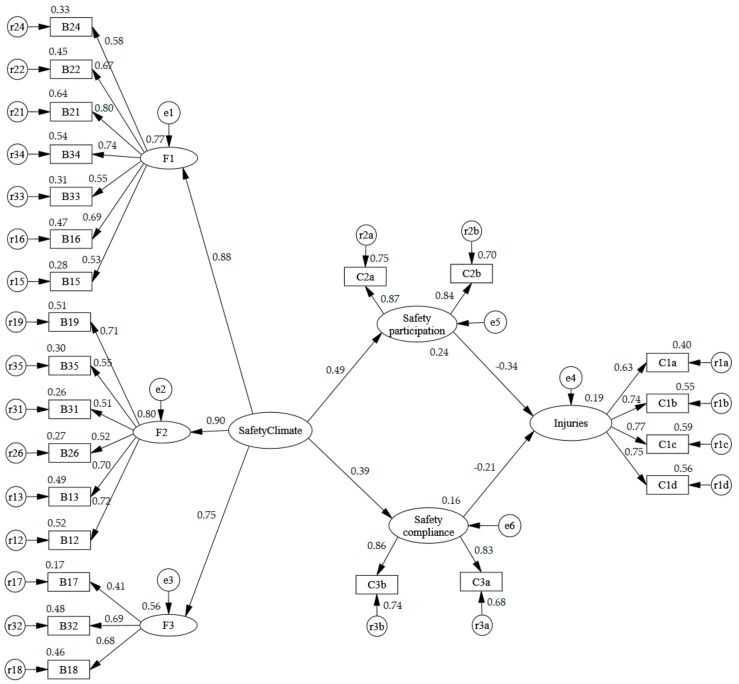
Structural equation model on ethnic minorities (EMs) with standardized path coefficients. Note: the description of each code in this figure is shown in Appendix A.

Prior to evaluation of the overall criteria, preliminary fit criteria were suggested to be used to examine the existence of anomalies [64]. Four types of preliminary fit criteria were scrutinized, and the results showed that: no negative error variances existed, as shown in Figure 2; no error variances were non-significant; no standardized factor loadings were smaller than 0.50 or greater than 0.95, (apart from the path from B17 “Sometimes it is necessary to take risks to get the job done” to F3, which almost reached 0.5); and no standard errors were extremely large.

Three categories of overall goodness-of-fit criteria comprising of ten indices suggested by previous studies and commonly used [65], were adopted to discuss the results of SEM, including (1) Absolute fit indices: root-mean-square error of approximation (RMSEA), root-mean-square residual (RMR), and adjusted goodness-of-fit index (AGFI); (2) Incremental fit indices: incremental fit index (IFI), Tucker–Lewis index (TLI), and the comparative fit index (CFI); (3) Parsimonious fit indices: parsimony normed-fit index (PNFI), parsimony goodness-of-fit index (PGFI), parsimony comparative fit index (PCFI), and ratio between chi-square and degree of freedom (χ^2^/df). The levels of acceptable fit for these ten measures, along with the values of these measures derived from this study, are presented in Table 4. Nine of ten indices satisfied the acceptance level, while the TLI was slightly smaller than 0.9. This indicates the high external quality of the model.

The fit of the internal structural model was taken into consideration, which refers to the significance test of paths. As shown in Table 5, all path coefficients were significant at a significance level of 0.05 or 0.001, indicating good internal structure fitness of the model. The composite reliability values of construct of safety participation, safety compliance, and near-misses and injuries were 0.843, 0.832, and 0.814, respectively, which are values higher than 0.7. This represents a satisfactory level of reliability of the internal indicators. The values of average variance extracted (AVE) for safety participation, safety compliance, near-misses and injuries were all higher than 0.5 (i.e., 0.729, 0.712, and 0.524, respectively). This indicates a satisfactory level of convergent validity of constructs. As for the evaluation of hierarchical models, values of composite reliability are higher than 0.7, indicating a satisfactory level of reliability of first-order constructs with the corresponding second-order construct.

All hypotheses in the model were tested based on the path analysis by SEM. Table 5 and Figure 2 present the results, including standardized path coefficients (*β*), the critical ratios (*t*), and the *p*-values. All six proposed hypotheses were supported. Safety climate has a significantly positive impact on safety participation (*β* = 0.491, *t* = 5.834, *p* < 0.001), supporting Hypothesis 1. Safety climate also shows a significantly positive correlation with safety compliance (*β* = 0.395, *t* = 4.845, *p* < 0.001), supporting Hypothesis 2. This indicates that one unit increase in safety climate leads to an approximately 0.49 and 0.39 unit increase in the safety participation and safety compliance of EMs, respectively. The former standardized path coefficient is higher than the latter, demonstrating Hypothesis 3 is supported. Support was found for Hypothesis 4 associating safety participation and near-misses and injuries of EMs (*β* = −0.342, *t* = −4.532, *p* < 0.001), and Hypothesis 5 linking safety participation and the near-misses and injuries of EMs (*β* = −0.207, *t* = −2.774, *p* < 0.05), with the former demonstrating the stronger relationship (supporting Hypothesis 6). This indicates that a one-unit increase in safety participation and safety compliance leads to an approximately 0.34- and 0.21-unit decrease in near-misses and injuries of EMs, respectively.

## 5. Discussion

EM workers are expected to continue to play a significant role in the construction industry in many countries. However, they are more vulnerable to occupational fatal and non-fatal accidents than their local counterparts. Empirical studies on the safety perceptions and safety performance of EM construction workers are rather limited. The current study examined the relationships among safety climate, safety behaviors, and safety outcomes of EM construction workers.

First, the results demonstrated that safety climate was positively related to safety behaviors (i.e., safety participation and safety compliance), and safety behaviors were negatively related to near-misses and injuries of EM construction workers. The finding on positive relationship between safety climate and safety behaviors is consistent with the empirical studies of Griffin and Neal [13] and Hon et al. [17]. They found that safety climate was positively related to safety behaviors in an Australian hospital and in the repair, maintenance, minor alteration, and addition (RMAA) sector in Hong Kong. In addition, the longitudinal studies of Neal and Griffin [45] in the Australian hospital and Pousette et al. [16] in the construction industry demonstrated that safety climate at a point in time predicts subsequent changes in safety behaviors, which in turn leads to changes in accidents. Indeed, a positive safety climate can motivate workers and increase their safety knowledge, leading to a reduction in violations of safety rules, regulations, and procedures of EM construction workers. In addition, according to the norm of reciprocity [66], if workers perceive that managerial and supervisorial staff highly value and are concerned for their well-being and safety, they are more likely to reciprocate through expanding their formal role into voluntary behaviors. Thus, a more positive safety climate improves safety compliance and safety participation of EM construction workers. Employers need to develop and maintain a supportive safety climate for EMs, taking into account the differences existing in the safety management of local workers and EM workers.

Second, the results of the present study showed that safety climate had a stronger relationship with safety participation than safety compliance. This finding is contrary to the argument of Hon et al. [17] that safety climate affects safety compliance more positively than the safety participation of local workers (standardized path coefficient = 0.66 and 0.22, respectively) in Hong Kong’s RMAA works. One explanation given by them is that local workers are inclined to perform by experience rather than safety rules and requirements. A positive safety climate tends to increase their level of safety compliance but is less likely to motivate them to participate voluntarily in safety activities. In this study, compared with safety participation, the level of safety compliance of EMs in Hong Kong’s construction industry is very high, as supported by the fact that the mean value of safety compliance is much higher than that of safety participation. This might be due to EMs’ characteristics and fear of losing their jobs. They normally are very willing to listen and learn safety requirements, follow instructions from supervisors, and comply with safety rules and procedures, and they seldom cut corners [30]. In this case, a positive safety climate tends to have a stronger positive influence on safety participation rather than safety compliance of EM construction workers. EM construction workers generally work in a multicultural environment where their co-workers, supervisors, or managers are local, and often face language and cultural barriers. Some EMs have low self-confidence, are very reserved, and seldom speak out [30]. These factors result in a low motivation to participate in extra safety activities. If they perceive a more positive safety climate, where management is concerned for their safety and encourages upward communication, they tend to be more likely to participate in safety activities. The results of this study also suggest that safety participation is more negatively related to near-misses and injures of EM construction workers than safety compliance. This may be explained by the fact that some voluntary behaviors of EMs, such as raising their concerns, promoting safety programs, and actively participating in meetings, can not only enhance their safety consciousness, but also help to improve the safety of the work environment for EMs.

Third, in the current study of EM construction workers, F1 *safety commitment, safety resources, and safety communication* and F2 *employee involvement and workmate’s influence* were found to have a more significant relationship with safety performance of EMs than F3 *safety rules, procedures and risks*. This finding is partly consistent with the argument of Choudhry et al. [67] that *management commitment and employee involvement* affect safety performance more significantly than *inappropriate safety procedures and work practices*. However, this study also emphasized the importance of the safety climate factor *safety resources, safety communication, and workmate’s influence* on the safety performance of EM construction workers, which shows the peculiarities in safety management for EMs. Safety resources suitable for EM construction workers (such as safety training and materials in their native languages, and availability of safety officers from the EM’s countries of origin) are lacking [30]. Adequate and appropriate safety trainings and materials should be provided to EMs. It is crucial to communicate safety requirements and instructions clearly and effectively to EM workers and ensure that they fully understand, considering they have higher level of safety compliance. Safety communication remains a big challenge for EMs as most of them have difficulties in understanding the predominant written languages used at construction sites in Hong Kong (i.e., Cantonese and English). Local management needs to improve intercultural communication and encourage upward communication of EM workers, so that EMs can feel free to express their safety concerns. Safety officers, supervisors, foremen, and gangers from EM origin countries need to be employed to act as bridges between management and EMs, due to the homophily theory that people tend to communicate with others who are similar to themselves, especially the same race and ethnicity [68]. Management should allow and encourage EMs to be involved in safety activities, such as the development of safety procedures, interventions, and safety planning, and in reporting site hazards and deficiencies in safety practice.

## 6. Conclusions

To conclude, this study empirically tested the hypothesized relationships of three safety climate factors on safety compliance, safety participation, and near-misses and injuries of EM construction workers with the help of SEM. The results supported that EM construction workers’ perceptions of safety climate are strongly positively related to their level of safety compliance and safety participation, and safety compliance and safety participation are negatively related to safety outcomes of EMs. Among three safety climate factors, F1 *safety management commitment, safety resources, and safety communication*, as well as F2 *employee’s involvement and workmate’s influence* were found to most positively affect safety behaviors of EMs.

This study provides empirical evidence regarding the influence of safety climate on safety behavior and safety outcomes for EM construction workers. A deeper understanding of the mechanism by which safety climate influences safety outcomes of EM construction workers could help employers to tailor safety and prevention programs for EM workers, which in turn would lead to fewer unsafe behaviors and lower accident rates. Interventions which focus on improving three safety climate dimensions can help to improve safety behaviors and decrease the number of accidents of EMs. This study also provides insights into safety management of EM construction workers for both researchers and practitioners in many areas where numerous EMs are employed, such as in the U.S., the UK, Australia, Singapore, Malaysia, and the Middle East.

This study has three main limitations. First, self-reported data on safety climate, safety behavior, and safety outcome may be subject to bias due to the reluctance of some EMs to report their actual perceptions. Second, this study only focused on the influence of safety climate on safety behaviors and safety outcomes for EM workers. As revealed by Christian et al. (2009), person-based factors (e.g., personality characteristics and job attitudes) and other situation factors (e.g., leadership) may also influence safety performance. Researchers in this field are advised to study other antecedents of safety behaviors and safety outcomes for EM construction workers apart from safety climate, in order to achieve a fuller picture of the mechanisms underlying the safety of EMs. Third, this study delimited the scope of research to EM workers and thus did not collect data from local workers at similar construction sites for comparison. It is recommended that further studies should be conducted to compare the safety behaviors of the EMs and those of the local workers at the same sites for direct comparison.

## Figures and Tables

**Table 1:** Construct measurement.

Constructs	Question Number	Supporting Literature
Safety climate	16	Chan et al. [53]
Safety participation	2	Neal and Griffin [45] and Hon et al. [17]
Safety compliance	2	Mohamed [54] and Hon et al. [17]
Near-misses and injuries	4	Hon et al. [17]

**Table 2:** Demographic information of the questionnaire survey respondents.

Demographic Variables		Number	Percent	Demographic Variables		Number	Percent
Nationality	Nepalese	233	80.6	Gender	Male	276	95.5
	Pakistani	56	19.4		Female	13	4.5
Age	20 or below	6	2.1	Employer	Contractor	195	67.5
	21–30	82	28.4		Subcontractor	94	32.5
	31–40	128	44.3	Work experience in the construction industry	<6 years	94	32.5
	41–50	67	23.2		6–10 years	116	40.1
	51 or above	6	2.1		11–15 years	39	13.5
Education level	Below primary	10	3.5		16–20 years	31	10.7
	Primary	68	23.5		>20 years	9	3.1
	Secondary	144	49.8	Length of service with the current company	<1 year	106	36.7
	Certificate	58	20.1		1–5 years	121	41.9
	Degree or higher	9	3.1		6–10 years	41	14.2
					>10 years	21	7.3

**Table 3:** Mean values and Cronbach’s alpha of constructs.

Construct	Mean	Number of Items	Cronbach’s Alpha
F1	3.81	7	0.831
F2	3.98	6	0.773
F3	3.28	3	0.618
Safety participation	3.64	2	0.844
Safety compliance	4.32	2	0.830
Near-misses and injuries	1.35	4	0.814

**Table 4:** Overall goodness-of-fit of the structural equation model. RMSEA: root-mean-square error of approximation; RMR: root-mean-square residual; AGFI: adjusted goodness-of-fit index; IFI: incremental fit index; TLI: Tucker–Lewis index; CFI: comparative fit index; PNFI: parsimony normed-fit index; PGFI: parsimony goodness-of-fit index; PCFI: parsimony comparative fit index.

Goodness-of-Fit Measures		This Study	Levels of Acceptable Fit
Absolute fit	RMSEA	0.057	<0.1
	RMR	0.057	<0.08
	AGFI	0.853	≥0.85
Incremental fit	IFI	0.910	≥0.9
	TLI	0.898	≥0.9
	CFI	0.909	≥0.9
Parsimonious fit	PNFI	0.738	>0.5
	PGFI	0.719	>0.5
	PCFI	0.807	>0.5
	χ^2^/df	1.936	<2

**Table 5:** Results of regression analysis.

Paths	Factor Loading	Standardized Factor Loading	S.E.	*p*	C.R.	Composite Reliability	AVE
Safety climate → Safety participation	1.180	0.491	0.202	***	5.834		
Safety climate → Safety compliance	0.442	0.395	0.091	***	4.845		
Safety participation → Injuries	−0.131	−0.342	0.029	***	−4.532		
Safety compliance → Injuries	−0.170	−0.207	0.061	**	−2.774		
Safety climate → F1	1	0.876	-	***	-	0.709	0.879
Safety climate → F2	0.922	0.895	0.121	***	7.600		
Safety climate → F3	0.729	0.748	0.145	***	5.042		
F1 → B21	1.124	0.797	0.115	***	9.814	0.839	0.431
F1 → B34	1.049	0.738	0.114	***	9.179		
F1 → B33	0.894	0.554	0.116	***	7.680		
F1 → B16	1.035	0.686	0.117	***	8.822		
F1 → B22	1.033	0.670	0.118	***	8.727		
F1 → B24	1	0.577	-	***	-		
F1 → B15	0.812	0.530	0.110	***	7.350		
F2 → B31	1.021	0.507	0.131	***	7.809	0.790	0.391
F2 → B26	0.963	0.516	0.119	***	8.074		
F2 → B13	1.133	0.702	0.110	***	10.346		
F2 → B35	1.017	0.547	0.119	***	8.563		
F2 → B19	1	0.714	-	***	-		
F2 → B12	1.374	0.721	0.126	***	10.888		
F3 → B17	1	0.407	-	***	-	0.626	0.369
F3 → B32	1.641	0.694	0.293	***	5.591		
F3 → B18	1.511	0.675	0.269	***	5.624		
Injuries → C1a	1	0.739	-	***	-	0.814	0.524
Injuries → C1b	1.134	0.631	0.118	***	9.610		
Injuries → C1c	1.068	0.768	0.119	***	8.971		
Injuries → C1d	1.043	0.750	0.106	***	9.834		
Safety participation → C2a	1	0.868	-	***	-	0.843	0.729
Safety participation → C2b	0.909	0.839	0.084	***	10.849		
Safety compliance → C3a	1	0.827	-	***	-	0.832	0.712
Safety compliance → C3b	1.137	0.860	0.147	***	7.758		

Note: S.E. = standardized error; ** *p* < 0.01, *** *p* < 0.001; C.R. = critical ratio (*t* value); AVE: average variance extracted. The description of each code in this figure is shown in Appendix A.

## Appendix A

Table A1 lists the 16 items used in this study to measure safety climate of EMs, and Table A2 provides the measures of safety behaviors and safety outcome of EMs, including safety participation, safety compliance, and near-misses and injures.

**Table A1:** Sixteen items measuring safety climate of EMs.

Code	Item
**Factor 1: Safety management commitment, safety resources, and safety communication**	
B24	Sufficient resources are available for health and safety here
B22	There are always enough people available to get the job done according to the health and safety procedures
B21	There are good communications here between management and workers about health and safety issues
B34	Staff are praised for completing jobs are reasonable
B33	My immediate boss often talks to me about health and safety matters on site
B16	There is good preparedness for emergency here
B15	The company encourages suggestions on how to improve health and safety
**Factor 2: Employee involvement and workmate’s influence**	
B19	I am clear about what my responsibilities are for health and safety
B35	Supervisors sometimes turn a blind eye to people who are not observing the health and safety procedures
B31	My workmates would react strongly against people who break health and safety procedures
B26	Work health and safety is not my concern
B13	All the people who work in my team are fully committed to health and safety
B12	People here always wear their personal protective equipment when they are supposed to
**Factor 3: Perception of safety rules, procedures, and risks**	
B17	Sometimes it is necessary to take risks to get the job done
B32	Not all the health and safety rules or procedures are strictly followed here
B18	I know that if I follow the safety rules or procedures, I will not get hurt

**Table A2:** Measures of safety behaviors and safety outcome of EMs.

Code	1. Number of Near-Misses and Occupational Injuries in the Last 12 Months (1 = Never; 2 = 1 time; 3 = 2–3 times; 4 = 4–5 times; 5 = Over 5 times)																							
C1a	(a) How many times have you exposed to a near miss incident of any kind at work?																	1	2	3		4		5
C1b	(b) How many times have you suffered from an accident/ injury of any kind at work, but did NOT require absence from work?																	1	2	3		4		5
C1c	(c) How many times have you suffered from an accident/ injury, which require absence from work NOT exceeding 3 consecutive days?																	1	2	3		4		5
C1d	(d) How many times have you suffered from an accident/injuries, which require absence from work exceeding 3 consecutive days?																	1	2	3		4		5
**Code**	**2. Safety Participation** **Please Circle the Most Appropriate Numbers. (1 = Never; 2 = Yearly; 3 = Monthly; 4 = Weekly; 5 = Daily)**																							
C2a	(a) How frequent do you put in extra effort to improve safety of the workplace (e.g., reminding co-workers about safety procedures at work)?																	1	2	3		4		5
C2b	(b) How frequent do you voluntarily carry out tasks or activities that help to improve workplace safety (e.g., attending safety meeting, receiving safety training)?																	1	2	3		4		5
**Code**	**3. Safety Compliance** **Please Circle on a Scale of 0–100% the Percentage of Time**																							
C3a	(a) You follow all of the safety procedures for the jobs that you perform.																							
	0	5	10	15	20	25	30	35	40	45	50	55	60	65	70	75	80	85	90		95		100	
C3b	(b) Your co-workers follow all of the safety procedures for the jobs that they perform.																							
	0	5	10	15	20	25	30	35	40	45	50	55	60	65	70	75	80	85	90		95		100	

## References

[1] Abdul-Rahman H., Wang C., Wood L.C., Low S.F. (2012). Negative impact induced by foreign workers: Evidence in Malaysian construction sector. Habitat Int. J..

[2] Department of Immigration and Citizenship (DIAC) (2009). Population Flows: Immigration Aspects: 2007–2008.

[3] Meardi G., Martín A., Riera M.L. (2012). Constructing uncertainty: Unions and migrant labour in construction in Spain and the UK. J. Ind. Relat..

[4] Census and Statistics Department (2013). 2011 Population Census Thematic Report: Ethnic Minorities.

[5] Chan A.P., Javed A.A., Wong F.K., Hon C.K. (2014). Improving Safety Communication of Ethnic Minorities in the Construction Industry of Hong Kong. ICCREM 2014: Smart Construction and Management in the Context of New Technology.

[6] Bust P.D., Gibb A.G.F., Pink S. (2008). Managing construction health and safety: Migrant workers and communicating safety messages. Saf. Sci..

[7] Tutt D., Pink S., Dainty A.R., Gibb A. (2013). ‘In the air’ and below the horizon: Migrant workers in UK construction and the practice-based nature of learning and communicating OHS. Constr. Manag. Econ..

[8] Centre for Corporate Accountability (CCA) (2009). Migrants’ Workplace Deaths in Britain.

[9] Goodrum P.M., Dai J. (2005). Differences in occupational injuries, illnesses, and fatalities among Hispanic and non-Hispanic construction workers. J. Constr. Eng. Manag..

[10] Al-Bayati A.J., Abudayyeh O., Fredericks T., Butt S.E. (2016). Reducing Fatality Rates of the Hispanic Workforce in the US Construction Industry: Challenges and Strategies. J. Constr. Eng. Manag..

[11] Hallowell M.R., Yugar-Arias I.F. (2016). Exploring fundamental causes of safety challenges faced by Hispanic construction workers in the US using photovoice. Saf. Sci..

[12] Chan A.P., Javed A.A., Wong F.K., Hon C.K., Lyu S. (2017). Evaluating the safety climate of ethnic minority construction workers in Hong Kong. J. Prof. Issues Eng. Educ. Pract..

[13] Griffin M.A., Neal A. (2000). Perceptions of safety at work: A framework for linking safety climate to safety performance, knowledge, and motivation. J. Occup. Health Psychol..

[14] Gillen M., Baltz D., Gassel M., Kirsch L., Vaccaro D. (2002). Perceived safety climate, job demands, and coworker support among union and nonunion injured construction workers. J. Saf. Res..

[15] Siu O.I., Phillips D.R., Leung T.W. (2004). Safety climate and safety performance among construction workers in Hong Kong: The role of psychological strains as mediators. Accid. Anal. Prev..

[16] Pousette A., Larsson S., Törner M. (2008). Safety climate cross-validation, strength and prediction of safety behaviour. Saf. Sci..

[17] Hon C.K., Chan A.P., Yam M.C. (2014). Relationships between safety climate and safety performance of building repair, maintenance, minor alteration, and addition (RMAA) works. Saf. Sci..

[18] Hickman J.S., Geller E.S. (2003). A safety self-management intervention for mining operations. J. Saf. Res..

[19] Cheng C.W., Leu S.S., Cheng Y.M., Wu T.C., Lin C.C. (2012). Applying data mining techniques to explore factors contributing to occupational injuries in Taiwan’s construction industry. Accid. Anal. Prev..

[20] Zou P.X., Sunindijo R.Y. (2013). Skills for managing safety risk, implementing safety task, and developing positive safety climate in construction project. Autom. Constr..

[21] Choudhry R.M., Fang D., Mohamed S. (2007). The nature of safety culture: A survey of the state-of-the-art. Saf. Sci..

[22] Hon C.K., Chan A.P., Yam M.C. (2012). Determining safety climate factors in the repair, maintenance, minor alteration, and addition sector of Hong Kong. J. Constr. Eng. Manag..

[23] Cox S., Cheyne A. (2000). Assessing safety culture in offshore environments. Saf. Sci..

[24] Seo D.C., Torabi M.R., Blair E.H., Ellis N.T. (2004). A cross-validation of safety climate scale using confirmatory factor analytic approach. J. Saf. Res..

[25] Dainty A.R., Gibb A.G., Bust P.D., Goodier C.I. (2007). Health, Safety and Welfare of Migrant Construction Workers in the South East of England.

[26] Ahonen E.Q., Porthé V., Vázquez M.L., García A.M., López-Jacob M.J., Ruiz-Frutos C., Ronda-Pérez E., Benach J., Benavides F.G. (2009). A qualitative study about immigrant workers’ perceptions of their working conditions in Spain. J. Epidemiol. Community Health.

[27] Santoso D.S. (2009). The construction site as a multicultural workplace: A perspective of minority migrant workers in Brunei. Constr. Manag. Econ..

[28] Roelofs C., Sprague-Martinez L., Brunette M., Azaroff L. (2011). A qualitative investigation of Hispanic construction worker perspectives on factors impacting worksite safety and risk. Environ. Health.

[29] Starren A., Hornikx J., Luijters K. (2013). Occupational safety in multicultural teams and organizations: A research agenda. Saf. Sci..

[30] Loosemore M., Lee P. (2002). Communication problems with ethnic minorities in the construction industry. Int. J. Proj. Manag..

[31] Chan A.P., Wong F.K., Hon C.K., Javed A.A., Lyu S. (2007). Construction safety and health problems of ethnic minority workers in Hong Kong. Eng. Constr. Archit. Manag..

[32] Mearns K., Yule S. (2009). The role of national culture in determining safety performance: Challenges for the global oil and gas industry. Saf. Sci..

[33] Mohamed S., Ali T.H., Tam W.Y.V. (2009). National culture and safe work behaviour of construction workers in Pakistan. Saf. Sci..

[34] Gao R., Chan A.P., Utama W.P., Zahoor H. (2016). Workers’ Perceptions of Safety Climate in International Construction Projects: Effects of Nationality, Religious Belief, and Employment Mode. J. Constr. Eng. Manag..

[35] Zohar D. (1980). Safety climate in industrial organizations: Theoretical and applied implications. J. Appl. Psychol..

[36] Flin R., Mearns K., O’Connor P., Bryden R. (2000). Measuring safety climate: Identifying the common features. Saf. Sci..

[37] Glendon A.I., Litherland D.K. (2001). Safety climate factors, group differences and safety behaviour in road construction. Saf. Sci..

[38] Olsen E. (2010). Exploring the possibility of a common structural model measuring associations between safety climate factors and safety behaviour in health care and the petroleum sectors. Accid. Anal. Prev..

[39] Wu C., Song X., Wang T., Fang D. (2015). Core dimensions of the construction safety climate for a standardized safety-climate measurement. J. Constr. Eng. Manag..

[40] Christian M.S., Bradley J.C., Wallace J.C., Burke M.J. (2009). Workplace safety: A meta-analysis of the roles of person and situation factors. J. Appl. Psychol..

[41] Borman W.C., Motowidlo S.J. (1997). Task performance and contextual performance: The meaning for personnel selection research. Hum. Perform..

[42] Neal A., Griffin M.A., Hart P.M. (2000). The impact of organizational climate on safety climate and individual behavior. Saf. Sci..

[43] Lu C.S., Yang C.S. (2010). Safety leadership and safety behavior in container terminal operations. Saf. Sci..

[44] Vinodkumar M., Bhasi M. (2010). Safety management practices and safety behaviour: Assessing the mediating role of safety knowledge and motivation. Accid. Anal. Prev..

[45] Neal A., Griffin M.A. (2006). A study of the lagged relationships among safety climate, safety motivation, safety behavior, and accidents at the individual and group levels. J. Appl. Psychol..

[46] Huang Y.H., Ho M., Smith G.S., Chen P.Y. (2006). Safety climate and self-reported injury: Assessing the mediating role of employee safety control. Accid. Anal. Prev..

[47] Gabbe B.J., Finch C.F., Bennell K.L., Wajswelner H. (2003). How valid is a self-reported 12 months sports injury history?. Br. J. Sports Med..

[48] Oliver A., Cheyne A., Tomas J.M., Cox S. (2002). The effects of organizational and individual factors on occupational accidents. J. Occup. Organ. Psychol..

[49] Zohar D. (2000). A group-level model of safety climate: Testing the effect of group climate on microaccidents in manufacturing jobs. J. Appl. Psychol..

[50] Hofmann D.A., Morgeson F.P., Gerras S.J. (2003). Climate as a moderator of the relationship between leader-member exchange and content specific citizenship: Safety climate as an exemplar. J. Appl. Psychol..

[51] Clarke S. (2006). The relationship between safety climate and safety performance: A meta-analytic review. J. Occup. Health Psychol..

[52] Cooper M.D., Phillips R.A. (2004). Exploratory analysis of the safety climate and safety behavior relationship. J. Saf. Res..

[53] Chan A.P., Wong F.K., Hon C.K., Lyu S., Javed A.A. (2017). Investigating ethnic minorities’ perceptions of safety climate in the construction industry. J. Saf. Res..

[54] Mohamed S. (2002). Safety climate in construction site environments. J. Constr. Eng. Manag..

[55] Nielsen K.J., Rasmussen K., Glasscock D., Spangenberg S. (2008). Changes in safety climate and accidents at two identical manufacturing plants. Saf. Sci..

[56] Nulty D.D. (2008). The adequacy of response rates to online and paper surveys: What can be done?. Assess. Eval. High. Educ..

[57] Moser C., Kalton G. (1980). Survey Methods in Social Investigation.

[58] Hair J.F., Anderson R.E., Babin B.J., Black W.C. (2010). Multivariate Data Analysis: A Global Perspective.

[59] Nunnally J.C., Bernstein I.H. (1994). Psychometric Theory.

[60] Pallant J. (2010). SPSS Survival Manual: A Step by Step Guide to Data Analysis Using SPSS.

[61] Ferketich S. (1990). Internal consistency estimates of reliability. Res. Nurs. Health.

[62] Robinson J.P., Shaver P.R., Wrightsman L.S. (1991). Criteria for scale selection and evaluation. Measures of Personality and Social Psychological Attitudes.

[63] Idvall E., Hamrin E., Unosson M. (2002). Development of an instrument to measure strategic and clinical quality indicators in postoperative pain management. J. Adv. Nurs..

[64] Bagozzi R.P., Yi Y. (1988). On the evaluation of structural equation models. J. Acad. Mark. Sci..

[65] Xiong B., Skitmore M., Xia B. (2015). A critical review of structural equation modeling applications in construction research. Autom. Constr..

[66] Gouldner A.W. (1960). The norm of reciprocity: A preliminary statement. Am. Soc. Rev..

[67] Choudhry R.M., Fang D., Lingard H. (2009). Measuring safety climate of a construction company. J. Constr. Eng. Manag..

[68] Katz N., Lazer D., Arrow H., Contractor N. (2005). The network perspective on small groups. Theories of Small Groups: Interdisciplinary Perspectives.

